# Breed-Associated Differences in Longissimus Dorsi Muscle Lipid Composition in Saanen Dairy Goat, Shaanbei White Cashmere Goat, and Tan Sheep, with Implications for Flavour Formation

**DOI:** 10.3390/foods15162777

**Published:** 2026-08-07

**Authors:** Cheng Pan, Tingting Yu, Yaming Chen, Longhui Liu, Yuzhu Guo, Guiao Wang, Wei Wang, Jun Luo

**Affiliations:** Key Laboratory of Animal Genetics, Breeding and Reproduction of Shaanxi Province, College of Animal Science and Technology, Northwest A&F University, Xianyang 712100, China; panchengswust@163.com (C.P.); yutingting202606@163.com (T.Y.); 17336368480@163.com (Y.C.); llh_hhhh@163.com (L.L.); 17382971211@163.com (Y.G.); wangguiao@126.com (G.W.); wangweixn007@nwsuaf.edu.cn (W.W.)

**Keywords:** lipidomics, UHPLC MS/MS, sphingolipids, glycerophospholipids, goat, sheep

## Abstract

Goat and sheep meat are highly regarded in the market for their unique flavor qualities. In this study, lipidomics technology was employed to compare the lipid differences in the longissimus dorsi muscle among 8-month-old Saanen dairy goats (DYG, *n* = 6), Shaanbei white cashmere goats (CAG, *n* = 6), and Tan sheep (TSH, *n* = 6). Electronic nose analysis was performed solely on the DYG samples, while the E-nose data for the CAG and TSH groups were referenced against previously published literature. The results indicated that aldehydes, ketones, methyl derivatives, and inorganic sulfides constituted the predominant stable volatile flavor compounds in the DYG muscle. Principal component analysis (PCA) and partial least square discriminant analysis (PLS-DA) revealed clear breed-dependent clustering and separation. Cluster analysis revealed that CAG and TSH groups exhibited highly similar lipid expression patterns, whereas the DYG group showed a distinct profile. Specifically, in the DYG group, SM (d18:1/20:3) and S1P (t17:0) were significantly increased, while most triglycerides (TG) were decreased and diglyceride DG (18:0/16:0) was increased. Additionally, multiple phosphatidylcholines (PC) and phosphatidylethanolamines (PE) were significantly reduced in the DYG group. This study revealed unique lipid signatures in the DYG group muscle compared with CAG and TSH groups, providing a theoretical basis for deciphering the lipid regulatory mechanisms underlying meat quality and flavor formation in goats and sheep.

## 1. Introduction

China is a prominent global producer of mutton. In 2023, the sheep and goat population reached 323 million head, with meat production totaling 5.31 million tonnes, reflecting a year-on-year increase of 1.3%. Meanwhile, per capita mutton consumption continued to rise, increasing from 3.97 kg in 2022 to 4.07 kg in 2023 [1]. As living standards improve, consumer demand for mutton quality has evolved, shifting from merely satisfying nutritional needs to seeking superior flavor characteristics [2,3,4]. Consequently, the enhancement of both the quality and flavor of mutton has become increasingly important.

Meat quality and flavor are influenced by a multitude of factors, including breed, sex, and age, with genetic factors playing a critical role in determining traits such as myofiber composition, fat deposition, and protein synthesis [5,6]. In Shaanxi Province, China, the livestock industry is well developed. The Shaanbei white cashmere goat (CAG) is renowned for its excellent cashmere production [7], while the Saanen dairy goat (DYG) is famous for its high milk yield [8]. Additionally, the Tan sheep (TSH) is a well-known breed in Ningxia, valued for both its high quality fur and meat [9]. These three livestock breeds exhibit distinct characteristics, leading to notable variations in the taste and flavor of their meat. Previous integrated multi-omics research has compared the flavor characteristics of meat from CAG and TSH [10]. The findings indicate that specific triglycerides, diglycerides, and dodecanoic acid play a regulatory role in meat flavor through three KEGG metabolic pathways: fat digestion and absorption, cholesterol metabolism, and lipid metabolism associated with atherosclerosis [10]. However, existing studies have not utilized lipidomics to compare the differences in muscle lipid profiles among dairy, cashmere, and meat-type livestock breeds. Therefore, this study utilized lipidomics to analyze the differences in muscle lipid profiles among TSH (meat-type), CAG (cashmere-type), and DYG (dairy-type), aiming to identify novel lipid biomarkers that regulate meat flavor.

Lipids are not only essential nutritional components of meat but also serve as key precursors for meat flavor [11]. Phospholipids, triglycerides, and free fatty acids present in muscle tissues generate a diverse array of volatile flavor compounds (e.g., aldehydes, ketones, alcohols) during heating and enzymatic hydrolysis, which directly affect the final flavor profile of the meat [12]. Therefore, an in-depth analysis of muscle lipid composition is crucial for understanding the underlying reasons for flavor differences in meat. In this study, lipidomics technology was employed to compare the lipid composition of muscle tissues from CAG, TSH, and DYG breeds. Primary objectives: To identify key differential lipid metabolites associated with inter-breed differences in muscle lipids, and to perform KEGG pathway enrichment analysis to excavate the potential metabolic pathways regulating these differential lipids. Secondary objective: To characterize the flavor characteristics of DYG muscle via electronic nose measurement. This study provides a theoretical foundation for elucidating the flavor formation mechanisms in these three breeds and offers scientific guidance for breed selection and flavor regulation in high-quality mutton production.

## 2. Materials and Methods

### 2.1. Ethics Statement

This study was approved by the Animal Ethics and Welfare Committee of Northwest A&F University (DK2022008, Approval Date: 9 November 2022). All procedures were conducted in accordance with the animal research guidelines established by Northwest A&F University. Furthermore, this study strictly adhered to the guidelines set forth in the Guide for the Care and Use of Laboratory Animals by the National Institutes of Health (NIH).

### 2.2. Animals and Sample Collection

Saanen dairy goats (DYG) were obtained from the Northwest A&F University Breeding Farm in Yangling, Shaanxi Province. Tan sheep (TSH) came from Yanchi County, Ningxia Hui Autonomous Region, Shaanbei, while cashmere goats (CAG) were obtained from Yulin City, Shaanxi Province. The nutritional composition of the experimental diet adhered to the agricultural industry standard of the People’s Republic of China (NY/T816-2004) [10]. All animals were housed uniformly indoors with free access to water. In this study, six 8-month-old male DYG, CAG, and TSH were slaughtered following standard procedures at a local abattoir. Prior to slaughter, all animals underwent a 24 h fasting period and were provided unlimited access to water. The abattoir facilities complied with the relevant requirements of China’s animal welfare and ethics guidelines. The longissimus dorsi muscle was excised from the right side of each carcass at the level of the 12th to 13th ribs. A single tissue sample was collected from this site per animal. All samples were immediately snap-frozen in liquid nitrogen and stored at −80 °C until analysis.

### 2.3. Electronic Nose Analysis

Volatile flavor characteristics of DYG group longissimus dorsi muscle were analyzed using a PEN3 electronic nose coupled with EDU3 enrichment and thermal desorption unit (AIRSENSE Analytics GmbH, Schwerin, Germany). The system was equipped with standard Tenax TA adsorbent tubes (50/100 mg) for selective trapping of medium/low volatile flavor compounds, and all instrument operations were controlled by WinMuster (v2.0.0.11) and TTD Terminal software (v1.01.11). The electronic nose is equipped with 10 distinct sensors to comprehensively capture the response patterns of various volatile compounds. The target analytes corresponding to each sensor are listed as follows: W1C (aromatic hydrocarbons), W5S (nitrogen oxides), W3C (amines), W6S (hydrogen), W5C (short-chain alkane aromatic components), W1S (methyl compounds), W2S (aldehydes and ketones), W1W (inorganic sulfides), W2W (sulph-chlor), and W3S (long-chain alkanes). A fresh meat sample weighing 3 g was put into a 50 mL container and equilibrated at a constant temperature of 20 °C for 2 h before detection. The entire process was conducted manually, with each sample being measured at least 6 times. The working parameters of the electronic nose were set as follows: sample preparation time was 5 s, cleaning time was 300 s, detection time was 60 s, and automatic zeroing time was 5 s, with both internal airflow and sample airflow rates set at 300 mL/min. During data analysis, sensor response signal values were selected from the detection phase between 45 and 50 s.

### 2.4. Lipid Extraction and Lipidomics Analysis Process

The lipid extraction method was performed as described by Zhang et al. [13]. Prior to lipid extraction, a mixture of deuterated lipid internal standards was spiked into each muscle homogenate to monitor the stability of the whole detection process. Six isotope-labeled internal standards covering multiple lipid classes were used, including three standards for ESI^+^ mode and three standards for ESI^−^ mode (Table 1). The brief procedure was as follows: A 50 mg aliquot of longissimus dorsi muscle tissue was added to 400 μL methyl tert-butyl ether (MTBE) and 80 μL methanol, followed by ultrasonic extraction for 30 min. After standing for 30 min, the sample was subjected to high-speed centrifugation. Then, 350 μL of the supernatant was dried in a vacuum concentrator and redissolved in 100 μL of reconstitution solution (acetonitrile: water = 1:1, *v*/*v*). After vortex mixing for 30 s, the mixture was ultrasonicated in an ice bath at a frequency of 40 kHz for 5 min. After centrifugation of the sample (13,000 rpm, 5 min, 4 °C), the supernatant was collected. An 80 μL portion of it was then loaded into a sample vial equipped with an insert for subsequent analytical procedures.

To evaluate the reproducibility of the entire analytical workflow, quality control (QC) samples were prepared by pooling equal volumes of metabolites from all samples. During instrumental analysis, one QC sample was inserted after every eight sample analyses. Chromatographic separation of lipids was performed using a Thermo UHPLC Vanquish Horizon (Thermo Fisher Scientific, Waltham, MA, USA) system equipped with an ACQUITY BEH C30 column (100 mm × 2.1 mm i.d., 1.7 μm; Waters, Milford, CT, USA). Mobile phase A consisted of an aqueous solution containing 50% acetonitrile, 0.1% formic acid, and 10 mmol/L ammonium acetate. Mobile phase B was composed of a mixture of acetonitrile, isopropanol, and water in a ratio of 10:88:2 (*v*/*v*/*v*), supplemented with 0.02% formic acid and 2 mmol/L ammonium acetate. The injection volume was set at 3 μL, and the column temperature was maintained at 40 °C. The samples were ionized using electrospray ionization (ESI), and mass spectrometry signals were obtained in both positive and negative ion scanning modes. The mass spectrometry parameters are shown in Table 2. After data acquisition, the raw LC-MS data were imported into LipidSearch software (v4.1, Thermo, Vacaville, CA, USA) for lipidomics data processing, including peak detection, baseline filtering, retention time alignment, integration, identification, and peak alignment. The major adduct types included [M+H]^+^, [M-H]^−^, [M+NH_4_]^+^, and [M+HCOO]^−^. This yielded a data matrix containing retention time, lipid name, peak intensity, mass-to-charge ratio, and other relevant information. Subsequently, feature peaks were identified using this software by matching MS and MS/MS spectral information against the metabolite database, with confirmatory identification based on an MS/MS spectral library. Putative identifications were assigned at MSI Level 2 (putative identification by MS/MS spectral matching). The precursor ion mass tolerance was set to 10.0 ppm, fragment ion mass tolerance was 25.0 ppm, retention time tolerance was 0.1 min, and the m-score cutoff was 2.0. Six deuterated isotope-labeled internal standards were applied to assess the analytical stability: the z-score of peak area for each internal standard was calculated, and all internal standards with z-scores within ±2 standard deviations indicated stable detection performance. The gradient elution procedure was as follows: mobile phase B was held at 35% at 0 min; linearly increased from 35% to 60% over 0–3 min; linearly raised from 60% to 85% during 3–10 min; linearly ramped up to 100% B from 10 to 12.5 min; maintained at 100% B for elution between 12.5 and 13.9 min; rapidly decreased back to 35% B from 13.9 to 14 min; and kept at 35% B from 14 to 16 min for column equilibration.

In the data preprocessing stage, the original feature matrix was initially filtered to exclude variables with a missing value rate exceeding 20% in any experimental group, thereby mitigating data sparsity. For the few remaining missing values after this filtering, minimum value imputation was employed for replacement. Following the handling of missing values, total sum normalization was conducted to standardize the response values of each sample, correcting for discrepancies in total abundance or total ion intensity among samples. Subsequently, the stability of the data was evaluated based on the coefficients of variation in features in quality control (QC) samples, leading to the exclusion of unstable features with a relative standard deviation (RSD) greater than 30%. Finally, to fulfill the assumptions of statistical tests and to minimize heteroscedasticity, the abundance values of the retained features were log10-transformed. The resulting data matrix was then utilized for subsequent multivariate statistical analyses and differential feature screening. Sample correlation analysis indicated reliable data repeatability (Figure S1).

### 2.5. Statistical Analysis

In this study, data are presented as mean ± standard deviation (SD). Bar graphs were generated using GraphPad Prism 9.0. One-way analysis of variance (ANOVA) was performed on each of the 84 lipid features via IBM SPSS Statistics 27 (IBM, Armonk, NY, USA) to compare lipid abundance across groups. The Benjamini–Hochberg (BH) FDR correction was applied to the raw *p*-values of all individual ANOVA tests to address multiple testing bias, and Tukey’s HSD test was used for post hoc pairwise comparisons between breeds. Principal component analysis (PCA), partial least squares discriminant analysis (PLS-DA), PLS-DA permutation test (200 permutations), heatmap analysis, cluster analysis, and KEGG pathway analysis were all performed on the Meiji Cloud platform. Differential features were selected if they simultaneously met VIP > 1 in the PLS-DA model and exhibited a *p*-value < 0.05. The differentially expressed metabolites obtained from the previous screening were mapped to KEGG pathways, where pathway enrichment significance was calculated by a hypergeometric test, followed by BH correction for multiple testing, and pathways with an adjusted *p*-value < 0.05 were considered significantly enriched.

## 3. Results

### 3.1. Electronic-Nose Odor Fingerprinting of Saanen Dairy Goat Muscle

**Figure 1:**
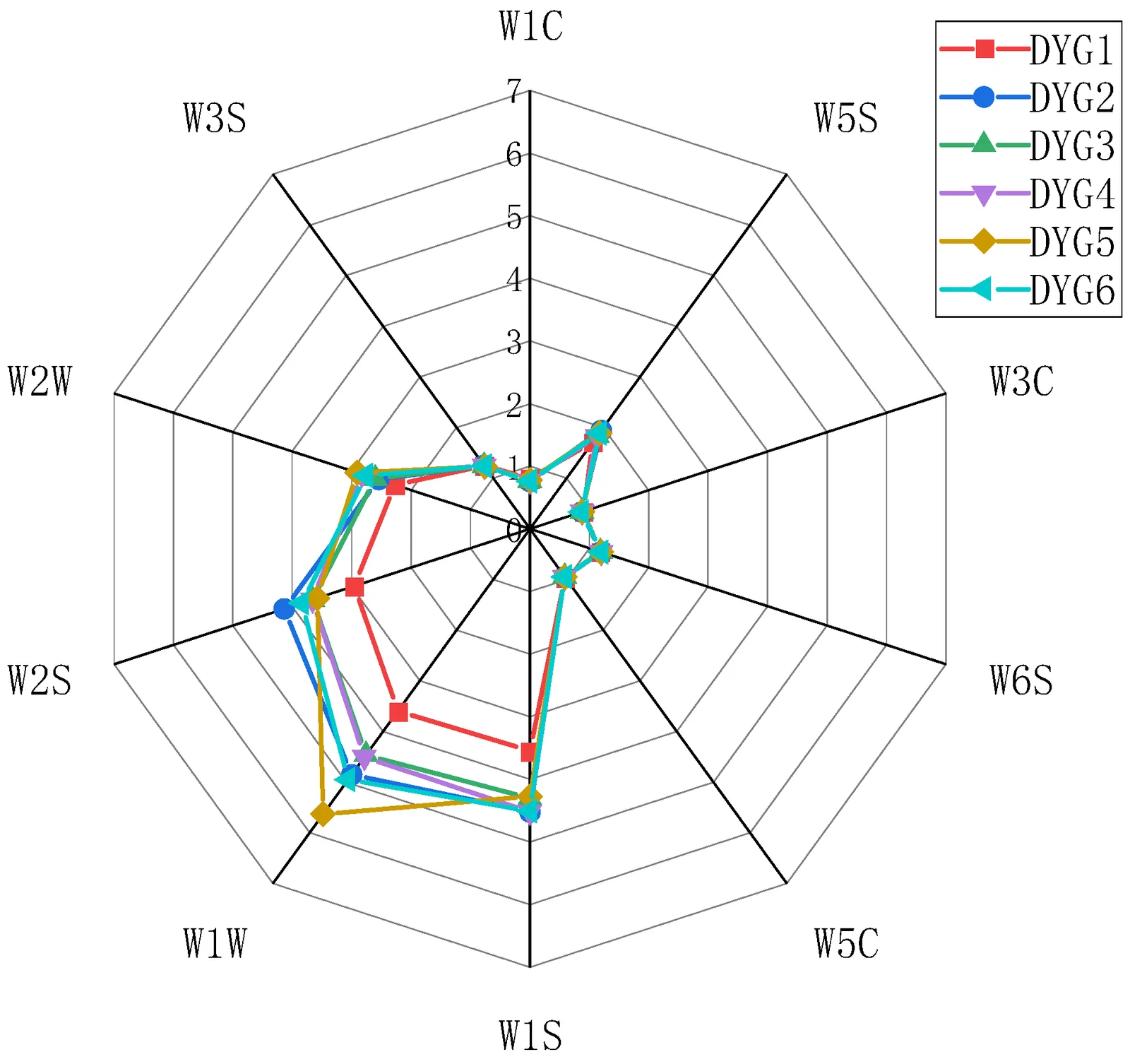
Electronic nose radar map of Saanen dairy goat meat (biological replicates n = 6). Data are expressed as mean ± standard deviation (SD). W1C (aromatic hydrocarbons), W5S (nitrogen oxides), W3C (amines), W6S (hydrogen), W5C (short-chain alkane aromatic components), W1S (methyl compounds), W2S (aldehydes and ketones), W1W (inorganic sulfides), W2W (sulph-chlor), and W3S (long-chain alkanes). No intergroup statistical comparisons were performed in this figure, as only DYG samples are displayed.

The electronic nose was employed to macro-measure the volatile components of Saanen dairy goat meat. The response values from several sensors, including W1C (aromatic hydrocarbons), W5S (nitrogen oxides), W3C (amines), W6S (hydrogen), W5C (short-chain alkane aromatic components), W1S (methyl compounds), W2S (aldehydes and ketones), W1W (inorganic sulfides), W2W (sulph-chlor), and W3S (long-chain alkanes), were analyzed. In the DYG group, the response values of the W1S, W2S, and W1W sensors exceeded 3 (Figure 1).

### 3.2. Annotation of CAG, TSH, and DYG Lipids

**Figure 2:**
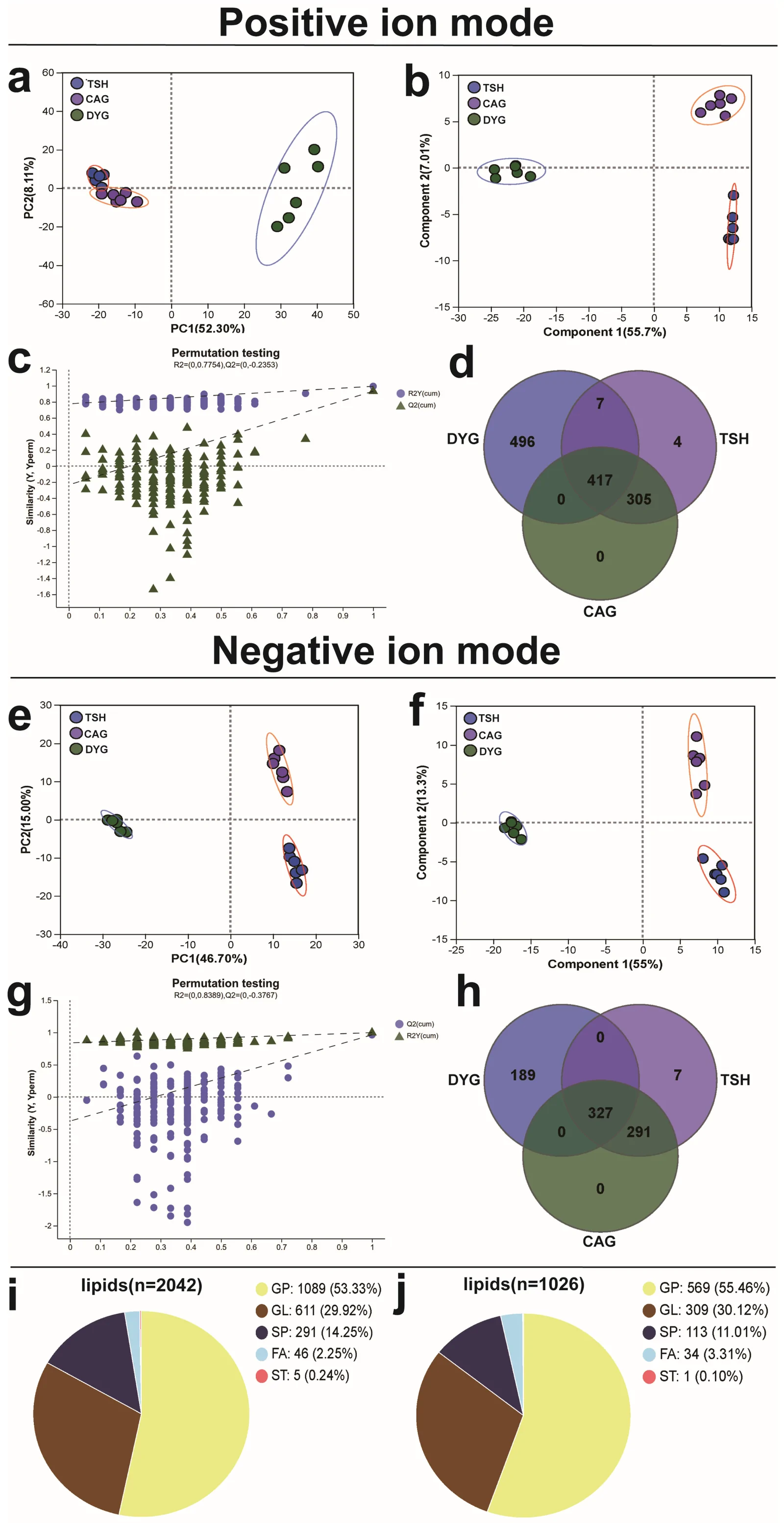
Annotation of CAG, TSH, and DYG lipids. (**a**,**b**,**e**,**f**) PCA and PLS-DA score plots. (**c**,**g**) Permutation test of PLS-DA. (**d**,**h**) Venn diagrams displaying the distribution of identified lipids across the three breeds in positive and negative ionization modes. (**i**) The pie chart shows the relative contributions (percentages) of FA, GP, GL, SP, and ST among the total of 2042 lipids identified across the three groups. (**j**) The pie chart illustrates the relative contributions (percentages) of FA, GP, GL, SP, and ST among the total of 1026 differential lipids identified across the three groups.

Lipidomics has enabled a systematic and comprehensive analysis of lipids in the longissimus dorsi muscle across CAG, TSH, and DYG breeds. Initially, principal component analysis (PCA) was employed to evaluate the overall variation and distribution characteristics of the dataset without predefined groupings [14]. In positive ion mode, the PCA model accounted for 60.41% of the total variance, with principal component 1 (PC1) and principal component 2 (PC2) contributing 52.30% and 8.11%, respectively (Figure 2a). Furthermore, partial least squares discriminant analysis (PLS-DA), a supervised dimensionality reduction and multivariate classification method [15], revealed clear clustering trends and distinct separation boundaries among the three breeds (Figure 2b). Permutation testing of the PLS-DA model verified the reliability of the dataset. Figure 2c presents permutation regression intercepts for positive-ion mode: R^2^Yintercept = 0.7754 and Qintercept^2^ = −0.2353. The intrinsic metrics of the original positive-ion model were R^2^Y = 0.994 and Q^2^ = 0.933 (Figure S2a). Venn diagram analysis showed that, in positive ion mode, 417 lipid metabolites were common to all three breeds, while 496 were unique to the DYG group, and 4 were unique to the TSH group (Figure 2d). In negative ion mode, the PCA model explained 61.70% of the total variance cumulatively (PC1: 46.70%, PC2: 15.00%) (Figure 2e). PLS-DA similarly revealed significant clustering and separation among the breeds (Figure 2f). Permutation testing of the PLS-DA model verified the reliability of the dataset. Figure 2g presents a permutation Q^2^ intercept of −0.3767 and an R^2^Y_intercept = 0.8389. The overall original model had R^2^Y = 0.997 and Q^2^ = 0.962 Figure S2b). Venn diagram analysis showed 327 common lipid metabolites across the three breeds, with 189 unique to the DYG group and 7 unique to the TSH group (Figure 2h). These results confirm that breed is a major determinant of muscle lipid composition. In total, 2042 lipid metabolites were identified and classified into five major categories: glycerophospholipids (GP), glycerolipids (GL), sphingolipids (SP), fatty acyls (FA), and sterol lipids (ST) (Figure 2i). Among these, GP accounted for the highest proportion (53.33%), followed by GL (29.92%) and SP (14.25%). Of the 2042 lipid metabolites, 1026 differential lipid metabolites were identified among the three breeds, with GP representing the largest proportion (55.46%) (Figure 2j). These results indicate that lipidomics can serve as an effective tool for analyzing meat quality.

### 3.3. Differential Lipids and Expression Patterns of CAG, TSH, and DYG

**Figure 3:**
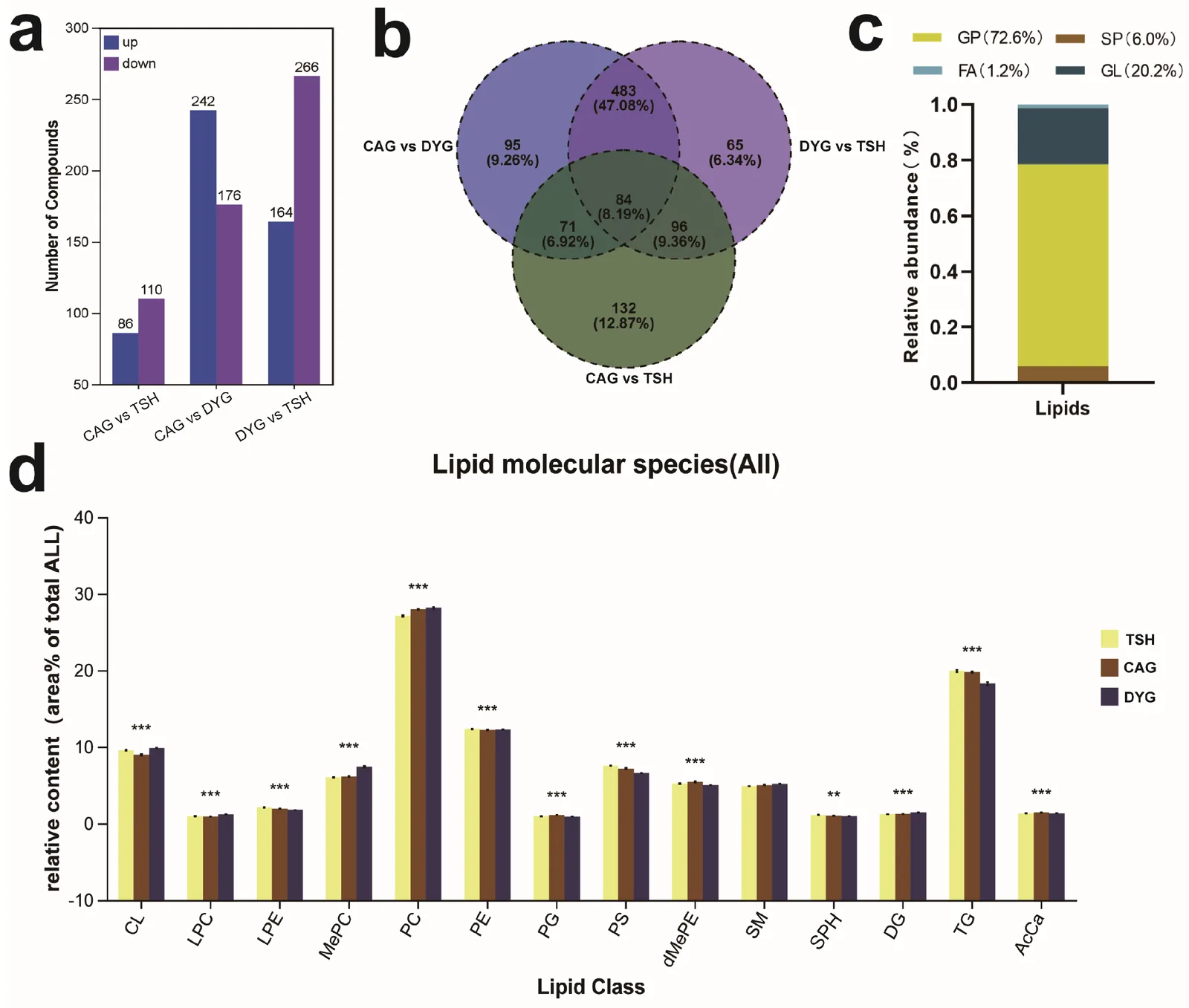
Differential lipid analysis. (**a**) Comparison of differential lipids among CAG, TSH, and DYG groups. (**b**) Venn diagrams displaying the distribution of identified lipids across the three breeds. (**c**) A percentage composition chart showing the classification of differential lipids common to the three groups. (**d**) Differential analysis of the classification of 84 shared differential lipids. ** *p* < 0.01, and *** *p* < 0.001.

This study investigated the effects of lipids on the flavor of the longissimus dorsi muscle in CAG, TSH, and DYG breeds, employing a PLS-DA model to analyze differential lipids (Figure 3a). A total of 196 differential lipids were identified between CAG and TSH groups, 418 between CAG and DYG, and 430 between DYG and TSH groups. Further analysis using a Venn diagram revealed that 84 differential lipids were common to all three breeds (Figure 3b). These differential lipids were classified into four major categories: GP (72.6%), SP (6.0%), FA (1.2%), and GL (20.2%) as shown in Figure 3c. Additionally, these lipid molecules were further categorized into 14 subclasses (Figure 3d), highlighting significant differences among the three breeds, including sphingosine (SPH), sphingomyelin (SM), triglycerides (TG), diglycerides (DG), acylcarnitines (AcCa), phosphatidylcholine (PC), cardiolipin (CL), and phosphatidylethanolamine (PE).

**Figure 4:**
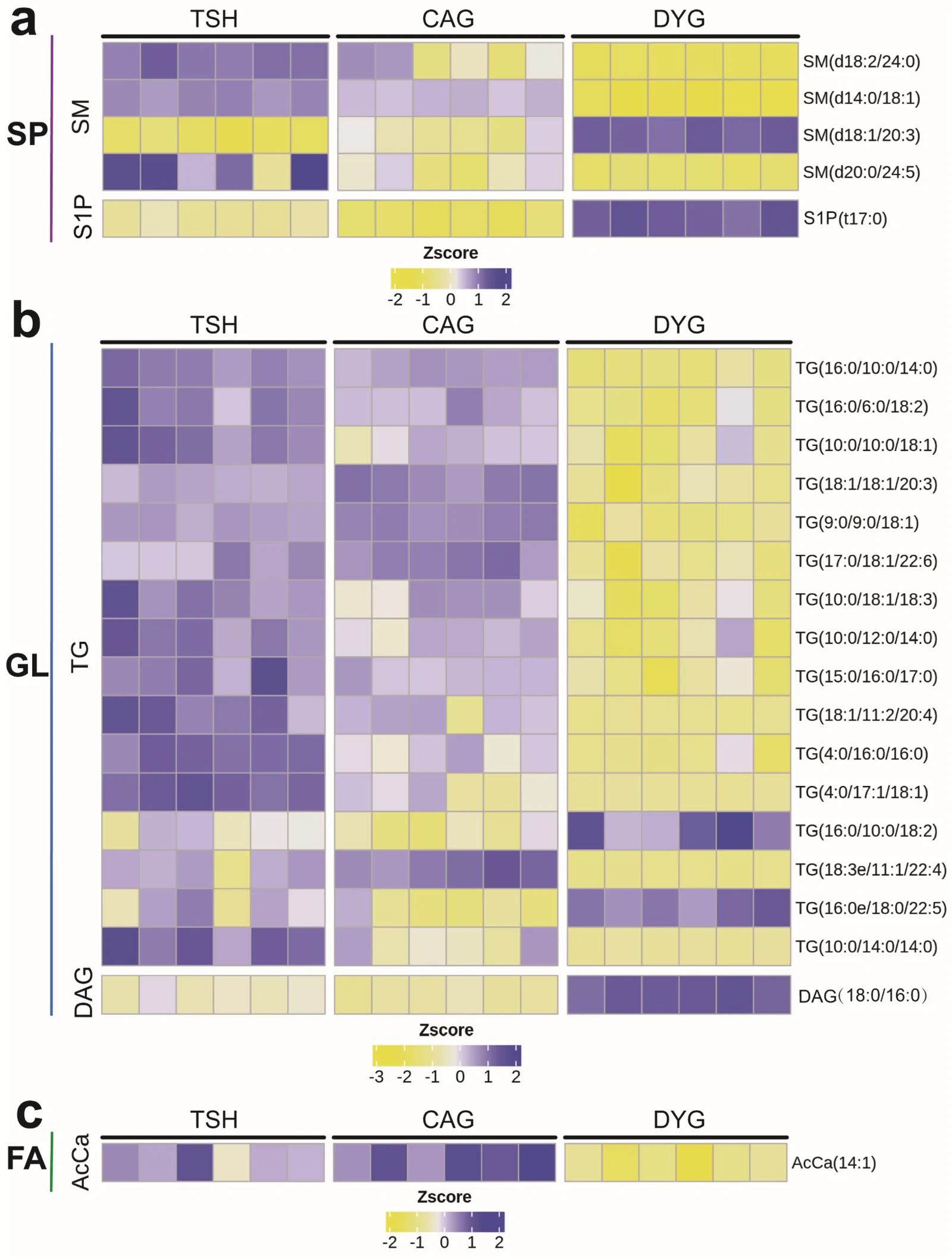
The clustering heatmap analysis shows the changes in lipid profiles among the three varieties. (**a**) SP. (**b**) GL. (**c**) FA.

**Figure 5:**
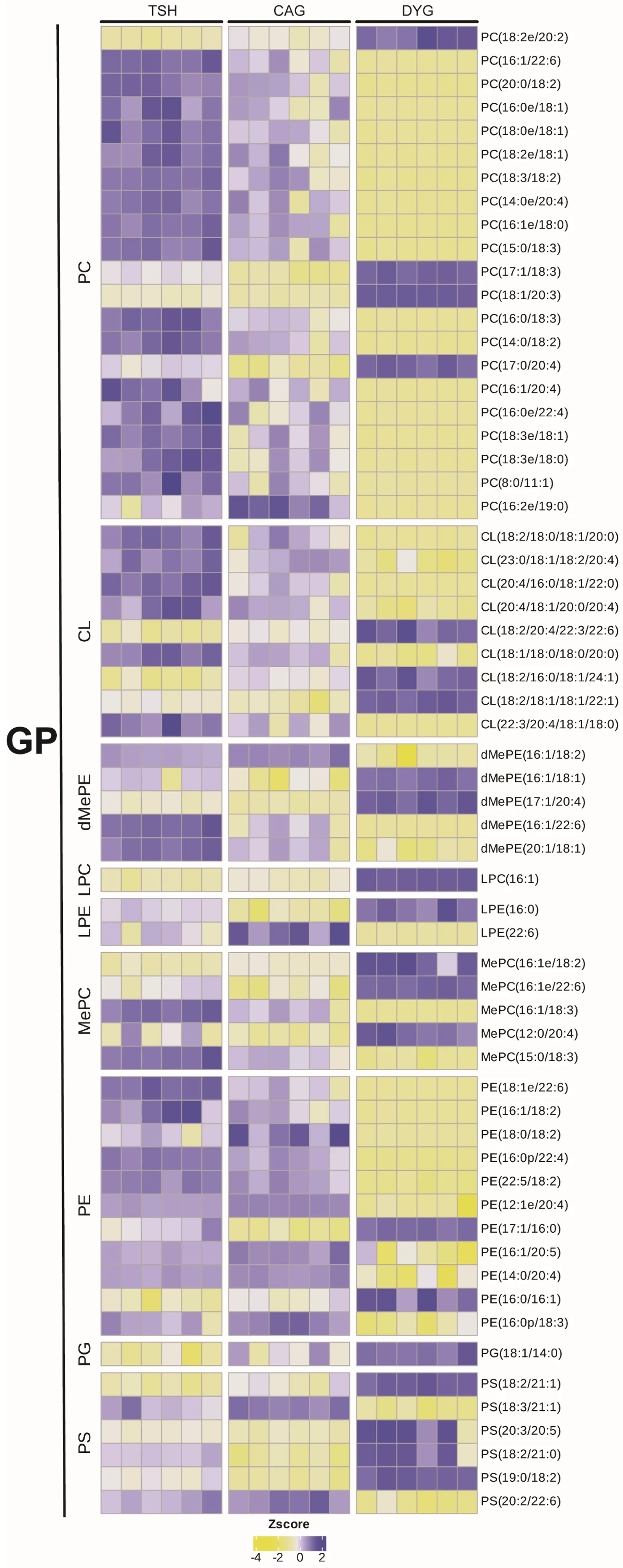
Clustering heatmap analysis shows the changes in GP lipid profiles among the three varieties.

To elucidate the expression patterns of 84 common differential lipids in the longissimus dorsi muscle across three breeds, a cluster analysis was conducted. The results indicated that the expression patterns of sphingomyelin (SM) and sphingosine phosphate (S1P) in the TSH and CAG groups were similar for the SP breed (Figure 4a). Additionally, SM (d18:1/20:3) and S1P (t17:0) were significantly elevated in the DYG group (Figure 4a, Table 3). For GL and FA, the expression patterns of triglycerides (TG), diacylglycerols (DG), and acylcarnitines (AcCa) in the TSH and CAG groups exhibited similarities, contrasting with those observed in DYG (Figure 4b,c). With the exception of TG (16:0/10:0/18:2) and TG (16:0e/18:0/22:5), all other TG lipids showed a significant decrease in the DYG group, while DG (18:0/16:0) was significantly increased in the same group (Figure 4b,c, Table 3). Furthermore, for glycerophospholipids (GP), the expression patterns of phosphatidylcholine (PC), cardiolipin (CL), and phosphatidylethanolamine (PE) in the TSH and CAG groups were more closely aligned and demonstrated significant differences from those in Saanen dairy goats. This was primarily reflected in the substantial decrease in the DYG group of lipids, including PC (16:1/22:6), PC (20:0/18:2), PC (16:0e/18:1), PC (18:0e/18:1), PE (18:1e/22:6), PE (16:1/18:2), PE (18:0/18:2), and PE (16:0p/22:4) (Figure 5, Table 3). In summary, the lipid metabolite expression patterns in the longissimus dorsi muscle of CAG and TSH groups are highly similar, while DYG exhibits a distinct expression pattern.

### 3.4. KEGG Pathway Analysis

**Figure 6:**
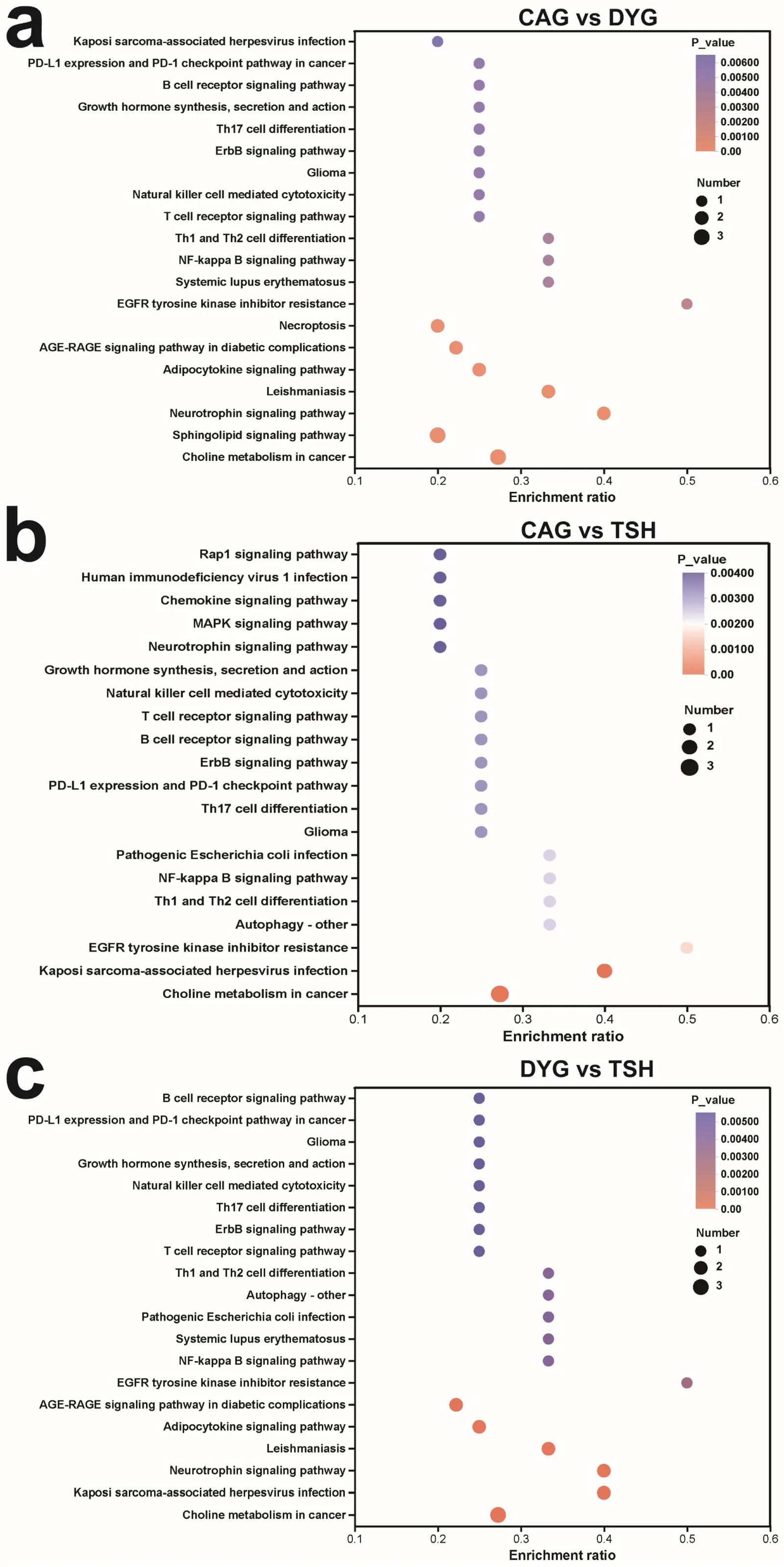
KEGG pathway analysis. (**a**–**c**) KEGG enrichment bubble plots. The x-axis represents the enrichment ratio, and the y-axis represents the KEGG pathways. The size of the bubbles indicates the degree of enrichment of metabolic compounds in the pathway, and the color of the bubbles represents the *p*-value for the significance of enrichment.

To further explore the functions of lipids in the CAG, TSH, and DYG groups, we conducted KEGG pathway analysis on all identified lipid metabolites. This analysis utilizes a hypergeometric distribution algorithm to evaluate the abundance of a selected set of metabolites, thereby quantifying metabolite concentrations. The results indicated that, compared to the CAG group, the differential lipids in the DYG group were predominantly enriched in the adipocytokine signaling pathway, leishmaniasis, neurotrophin signaling pathway, sphingolipid signaling pathway, and choline metabolism in cancer (Figure 6a). In contrast, the differential lipids in the TSH group, relative to the CAG group, were primarily enriched in Th1 and Th2 cell differentiation, autophagy—other, EGFR tyrosine kinase inhibitor resistance, Kaposi sarcoma-associated herpesvirus infection, and choline metabolism in cancer (Figure 6b). Furthermore, when comparing the DYG and TSH groups, the differential lipids in the TSH group were mainly enriched in the adipocytokine signaling pathway, leishmaniasis, neurotrophin signaling pathway, Kaposi sarcoma-associated herpesvirus infection, and choline metabolism in cancer (Figure 6c).

## 4. Discussion

As consumer demand for high-quality, healthy small ruminant meat continues to grow, this type of meat is increasingly recognized as a suitable and nutrient-rich protein source in Chinese cuisine. Consequently, the popularity of small ruminant meat in China is steadily rising [16,17]. There is an urgent need for effective methods to improve the meat quality and nutritional value of small ruminant meat, which holds significant implications for both the industry and consumers. The findings of this study suggest potential regional differences in the lipid composition of the longissimus dorsi muscle in sheep and goats from various parts of China, providing a theoretical reference for enhancing the meat quality and flavor of small ruminant meat through lipid regulation.

Goat and sheep meat exhibit distinct aromas and volatile characteristics that vary among different breeds [18]. In recent years, electronic noses have been extensively employed for the flavor analysis of goat and sheep meat [10,19]. Li et al. [20] conducted a flavor study on samples from the Taihang Mountain goat and Huai Bei goat, revealing significant differences in the concentrations of volatile compounds between these goat meat samples, particularly concerning nitrogen oxides, methylated compounds, aldehydes, and ketones. This study found that in the DYG group, the response values of the W1S, W2S, and W1W sensors were all greater than 3, indicating that aldehydes, ketones, methylated compounds, and inorganic sulfides are the primary and stable volatile components in the meat of Saanen dairy goats.

Lipids play a crucial role in the formation of meat flavor [21,22]. Li et al. [23] utilized lipidomics to investigate the effect of castration on the quality of the psoas major muscle in lambs, discovering that 224 lipid molecules increased in the psoas major muscle of castrated lambs. These changes in lipid molecules resulted in a richer profile of flavor precursors, thereby contributing to the improved quality of small ruminant meat. Zhang et al. [24] employed multi-omics techniques to analyze differences in the meat quality among Chongming white goats, Yichang white goats, and Xiaoxian white goats, revealing variations in the metabolism of amino acids, purines, glycerophospholipids, and glycerolipids among the three breeds. In this study, lipidomics was used to compare the lipid composition of the longissimus dorsi muscle among CAG, TSH, and DYG groups. A total of 1026 differential lipids were identified, of which 84 were common across three breeds. Among these common differential lipids, GP accounted for the highest proportion, followed by GL, SP, and FA. Consistent with the findings of Zhang et al. [24], these results suggest that the meat flavor of small ruminants may differ among breeds from different regions of China. Notably, the CAG group contributed no unique lipid features in either positive or negative ion mode, whereas the DYG group yielded 496 features in positive ion mode and 189 in negative ion mode. Combined with the QC quality control data, this asymmetry in detection coverage can be attributed to differences in lipid metabolism between the two breeds.

Glycerophospholipids (GP) are important structural and functional components of biological membranes, significantly contributing to the maintenance of cellular integrity [25]. They primarily comprise phosphatidylcholine, phosphatidylethanolamine, phosphatidylglycerol, phosphatidylinositol, phosphatidylserine, and phosphatidic acid [26]. Research has shown that GP possess notable therapeutic potential, including the inhibition of influenza A virus infection and the reduction in inflammatory cell infiltration [27]. Furthermore, studies indicate that GP are important components of small ruminant meat, closely associated with its quality and nutritional value [28]. Li et al. [29] reported that the GP composition in the muscle of goats varies with altitude. For example, compared to the Jianzhou big-eared goat, glycerophospholipids such as PE (16:0_20:5), PE (20:5_16:1), PE (17:1_22:6), and PS (18:2_20:5) were significantly upregulated in Xizang goat meat [29]. Another study revealed that specific phosphatidylcholines (e.g., PC 18:1e, PC 18:2e) serve as key markers distinguishing grazing from housed small ruminants including both sheep and goats [30]. These results indicate that lipids such as PC and PE not only serve as indicators of dietary and altitude effects but also play an important role in the flavor development of small ruminant meat. This aligns with our observation that GP are among the most significant differential lipids across the three breeds, suggesting their potential contribution to flavor enhancement. Therefore, this study hypothesizes that PC and PE play a critical role in shaping the flavor profile of small ruminant meat. Therefore, we speculate that these lipids may play a role in meat quality and flavor formation.

Glycerolipids (GL) are composed of a glycerol backbone and fatty acids, and are primarily classified into monoacylglycerols, diacylglycerols, and triacylglycerols [31]. Among these, triacylglycerols (TG) contain one fatty acid bound at each of the sn-1, sn-2, and sn-3 positions, serving as a crucial energy reservoir for cellular growth and metabolism [32]. Lipid peroxidation is widely recognized as a significant contributor to the deterioration of meat quality, adversely affecting the color and nutritional value of meat products while potentially generating toxic and harmful substances. Studies have established a direct link between lipid oxidation and lipid molecules, with the TG and phospholipids being the primary lipid molecules involved in lipid oxidation in meat products [33,34]. For example, Xu et al. [35] reported that during the late stage of meat storage, the downregulation of TG (especially TG involved in saturated fatty acid formation) may affect the aging process of small ruminant meat. In our study, we observed that TG content was highest in the TSH group, lowest in the DYG group, and intermediate in the CAG group (Figure 3d), suggesting that TG can be used as a vital indicator to assess meat quality and flavor.

In addition to GP and GL, several sphingolipids (SP) have been identified in the meat of small ruminants from the three breeds studied. SP mainly includes sphingomyelin, ceramide, and lysosphingomyelin. Their anti-inflammatory properties and potential in preventing colon cancer have garnered significant attention [36,37]. NORRIS et al. [38] demonstrated that sphingomyelin derived from eggs alleviated weight gain, hyperglycemia, and hyperlipidemia in mice subjected to a high-fat diet. Furthermore, sphingomyelin and its metabolites are essential components of the myelin sheath in the central nervous system [39,40]. Therefore, this study hypothesizes that dietary consumption of small ruminant meat may positively influence human health. Research indicates that specific phospholipid molecules regulate intramuscular fat (IMF) deposition by modulating lipid droplet formation [41]. Levels of SM (d40:1), SM (d42:1), and SM (d42:4) were significantly increased in the high IMF group of Hu sheep [42]. In this study, the levels of SM (d18:2/24:0), SM (d14:0/18:1), and SM (d20:0/24:5) were found to be highest in the TSH group and lowest in the DYG group. Therefore, this study hypothesizes that the longissimus dorsi muscle of Tan sheep possesses a higher IMF content.

KEGG analysis revealed that the differential lipids between group DYG and group CAG were significantly enriched in the sphingolipid signaling pathway, which is directly associated with the physiological functions of skeletal muscle [43]. The adipokine signaling pathway was also enriched in both the DYG vs. TSH and DYG vs. CAG comparison groups. Previous studies have verified that the adipokine signaling pathway is closely linked to the regulation of IMF deposition and serves as a pivotal signaling pathway governing the development of meat quality and flavor profiles [44,45]. Accordingly, this study hypothesizes that the adipokine signaling pathway may interact with the lipid metabolism regulatory networks in goat and sheep muscle, ultimately altering IMF deposition. However, the precise underlying molecular mechanisms remain to be further clarified. In addition, pathways including Kaposi sarcoma-associated herpesvirus infection, glioma, Th1/Th2 cell differentiation, leishmaniasis and systemic lupus erythematosus were also enriched in this study, which highlights the universal limitations of standard KEGG enrichment analysis when applied to lipidomics datasets.

The present study established correlations between lipid molecules and meat flavor in goats and sheep raised for different production purposes. Nevertheless, several limitations exist in this research. First, key meat quality indicators including intramuscular fat content, fatty acid composition and n-6/n-3 polyunsaturated fatty acid ratio were not determined. The analysis of flavor and fat deposition patterns was only based on general lipid classes without quantitative fatty acid data, which limits the reliability of relevant deductions. Second, characteristic flavor compounds specific to ovine and caprine meat such as branched-chain fatty acids and skatole were not quantified, and the metabolic linkage between differential lipids and these signature flavor substances was not elucidated. Third, all experimental animals were raised in their respective natural feeding environments and were not matched by body weight. The observed intergroup differences are subject to breed-by-environment interactions, making it difficult to attribute metabolic variations solely to genetic or environmental factors. Fourth, the sample size of six animals per breed is relatively small for high-dimensional lipidomic profiling containing 2042 annotated lipid features and multiple pairwise statistical comparisons. Without a formal power calculation to support the adequacy of n = 6, the current work should be considered exploratory and primarily serves to generate hypotheses. Further validation experiments with expanded sample cohorts are required to verify the lipid-related differences identified in this study. In addition, future research should supplement targeted quantification of fatty acids and characteristic flavor compounds to consolidate the evidence chain linking lipid metabolism to meat flavor.

## 5. Conclusions

This study identifies key lipids associated with flavor differences in goat and sheep meat among various production purposes. Lipidomic profiling identified 1026 differential lipids, with 84 shared by goat and sheep meat. The results indicate that PC, PE, and TG may contribute to the flavor characteristics of meat. Furthermore, SP could be linked to IMF deposition. This study provides an important understanding of the lipidomic processes that potentially participate in meat flavor formation and establishes a scientific foundation for future flavor optimization research in the meat industry.

## Figures and Tables

**Table 1:** Basic information of internal standards.

Formula	ExactMass	Mode	*m*/*z*
C23H39D7NO7P	486.3451	Positive	487.3524
C38H67D7NO8P	710.5591	Positive	711.5664
C51H89D7O6	811.7646	Positive	829.7985
C23H39D7NO7P	486.3451	Negative	485.3379
C38H67D7NO8P	710.5591	Negative	709.5519
C39H68D7O10P	741.5537	Negative	740.5464

**Table 2:** The mass spectrometry parameters.

Description	Parameter
Scan type (*m*/*z*)	200–2000
Sheath gas flow rate (psi)	60
Aux gas flow rate (psi)	20
Aux gas heater temp (°C)	370
IonSpray Voltage Floating (ESI^+^) (V)	+3000
IonSpray Voltage Floating (ESI^−^) (V)	−3000
Normalized collision energy (%)	20–40–60

**Table 3:** Analysis of 84 shared differential lipids.

Lipid	Class	DYG	TSH	CAG	FDR
AcCa (14:1)	AcCa	7.92 ± 0.17 ^b^	8.51 ± 0.20 ^a^	8.76 ± 0.18 ^a^	0.0003295
CL (18:1/18:0/18:0/20:0)	CL	8.71 ± 0.11 ^a^	7.55 ± 0.13 ^b^	6.69 ± 0.27 ^c^	5.88 × 10^−7^
CL (18:2/18:0/18:1/20:0)		8.63 ± 0.09 ^a^	6.86 ± 0.41 ^b^	5.98 ± 0.66 ^c^	5.29 × 10^−5^
CL (20:4/18:1/20:0/20:4)		8.40 ± 0.10 ^a^	6.68 ± 0.10 ^b^	5.57 ± 0.29 ^c^	4.25 × 10^−8^
CL (18:2/20:4/22:3/22:6)		6.27 ± 0.02 ^c^	7.32 ± 0.13 ^a^	6.73 ± 0.14 ^b^	8.61 × 10^−6^
CL (18:2/18:1/18:1/22:1)		5.77 ± 0.02 ^c^	6.98 ± 0.13 ^a^	6.40 ± 0.32 ^b^	5.33 × 10^−6^
CL (20:4/16:0/18:1/22:0)		5.65 ± 0.02 ^c^	6.75 ± 0.12 ^a^	6.30 ± 0.24 ^b^	5.13 × 10^−6^
CL (23:0/18:1/18:2/20:4)		5.33 ± 0.02 ^c^	6.88 ± 0.11 ^a^	6.03 ± 0.35 ^b^	9.38 × 10^−7^
CL (22:3/20:4/18:1/18:0)		5.31 ± 0.02 ^c^	6.44 ± 0.26 ^a^	5.85 ± 0.31 ^b^	0.0002111
CL (18:2/16:0/18:1/24:1)		5.27 ± 0.02 ^c^	6.30 ± 0.33 ^a^	5.82 ± 0.33 ^b^	0.0007152
DG (18:0/16:0)	DG	8.58 ± 0.06 ^a^	7.81 ± 0.11 ^b^	7.66 ± 0.07 ^c^	1.87 × 10^−7^
dMePE (16:1/18:1)	dMePE	8.80 ± 0.09 ^a^	7.49 ± 0.10 ^b^	7.12 ± 0.34 ^c^	2.94 × 10^−7^
dMePE (16:1/18:2)		6.12 ± 0.02 ^c^	7.45 ± 0.11 ^a^	6.70 ± 0.25 ^b^	1.95 × 10^−6^
dMePE (16:1/22:6)		5.46 ± 0.02 ^c^	7.31 ± 0.21 ^a^	6.45 ± 0.41 ^b^	8.38 × 10^−6^
dMePE (17:1/20:4)		5.42 ± 0.02 ^c^	6.06 ± 0.38 ^b^	7.07 ± 0.45 ^a^	0.0004382
dMePE (20:1/18:1)		4.45 ± 0.02 ^c^	5.20 ± 0.35 ^b^	6.10 ± 0.58 ^a^	0.0007181
LPC (16:1)	LPC	7.16 ± 0.26 ^a^	6.00 ± 0.60 ^b^	5.40 ± 0.22 ^c^	1.84 × 10^−5^
LPE (16:0)	LPE	6.01 ± 0.02 ^c^	7.26 ± 0.26 ^a^	6.68 ± 0.33 ^b^	0.0001357
LPE (22:6)		4.54 ± 0.02 ^c^	6.17 ± 0.42 ^a^	5.22 ± 0.47 ^b^	0.0003703
MePC (16:1/18:3)	MePC	9.36 ± 0.77 ^a^	8.18 ± 0.06 ^b^	7.96 ± 0.10 ^c^	0.003088
MePC (12:0/20:4)		9.36 ± 0.77 ^a^	8.53 ± 0.14 ^b^	7.41 ± 0.32 ^c^	0.0007316
MePC (15:0/18:3)		9.14 ± 0.11 ^a^	7.86 ± 0.14 ^b^	7.28 ± 0.19 ^c^	2.26 × 10^−7^
MePC (16:1e/18:2)		8.65 ± 0.17 ^a^	7.01 ± 0.40 ^c^	7.74 ± 0.47 ^b^	0.0003398
MePC (16:1e/22:6)		8.16 ± 0.06 ^a^	7.23 ± 0.10 ^c^	7.44 ± 0.15 ^b^	1.58 × 10^−6^
PC (16:0/18:3)	PC	8.71 ± 0.02 ^c^	9.98 ± 0.06 ^a^	9.65 ± 0.17 ^b^	3.33 × 10^−8^
PC (18:0e/18:1)		9.78 ± 0.18 ^a^	8.42 ± 0.21 ^c^	8.93 ± 0.09 ^b^	2.47 × 10^−5^
PC (16:1e/18:0)		10.24 ± 0.41 ^a^	8.97 ± 0.08 ^b^	9.16 ± 0.06 ^b^	0.0006798
PC (17:0/20:4)		7.84 ± 0.36 ^b^	8.87 ± 0.03 ^a^	9.04 ± 0.02 ^a^	2.12 × 10^−5^
PC (16:0e/18:1)		9.17 ± 0.17 ^a^	8.06 ± 0.13 ^c^	8.35 ± 0.07 ^b^	2.47 × 10^−5^
PC (14:0/18:2)		7.59 ± 0.10 ^c^	9.12 ± 0.13 ^a^	8.85 ± 0.23 ^b^	2.96 × 10^−7^
PC (18:2e/18:1)		7.21 ± 0.50 ^b^	8.39 ± 0.08 ^a^	8.63 ± 0.11 ^a^	0.0005814
PC (18:1/20:3)		9.77 ± 0.03 ^a^	7.41 ± 0.13 ^b^	7.04 ± 0.09 ^c^	4.85 × 10^−9^
PC (14:0e/20:4)		9.58 ± 0.05 ^a^	7.10 ± 0.19 ^c^	7.44 ± 0.17 ^b^	1.74 × 10^−7^
PC (16:0e/22:4)		7.00 ± 0.76 ^b^	8.20 ± 0.08 ^a^	8.54 ± 0.20 ^a^	0.003444
PC (16:2e/19:0)		8.09 ± 0.18 ^b^	8.83 ± 0.15 ^a^	9.06 ± 0.16 ^a^	9.19 × 10^−5^
PC (18:2e/20:2)		9.49 ± 0.21 ^a^	8.08 ± 0.08 ^c^	8.41 ± 0.11 ^b^	1.14 × 10^−5^
PC (15:0/18:3)		7.94 ± 0.12 ^c^	9.12 ± 0.12 ^a^	8.59 ± 0.16 ^b^	2.66 × 10^−6^
PC (16:1/20:4)		9.43 ± 0.07 ^a^	8.56 ± 0.35 ^b^	7.63 ± 0.23 ^c^	1.35 × 10^−5^
PC (18:3e/18:0)		8.97 ± 0.29 ^a^	7.82 ± 0.27 ^b^	8.13 ± 0.13 ^b^	0.001118
PC (20:0/18:2)		7.57 ± 0.28 ^b^	8.72 ± 0.19 ^a^	8.41 ± 0.23 ^a^	0.0003748
PC (8:0/11:1)		7.72 ± 0.13 ^c^	8.31 ± 0.16 ^b^	8.55 ± 0.07 ^a^	2.56 × 10^−5^
PC (18:3e/18:1)		7.13 ± 0.73 ^b^	8.46 ± 0.07 ^a^	8.64 ± 0.11 ^a^	0.003488
PC (18:3/18:2)		5.37 ± 0.46 ^c^	7.65 ± 0.20 ^a^	6.56 ± 0.52 ^b^	5.37 × 10^−5^
PC (17:1/18:3)		4.85 ± 0.02 ^c^	6.77 ± 0.16 ^a^	6.04 ± 0.52 ^b^	1.72 × 10^−6^
PC (16:1/22:6)		6.61 ± 0.05 ^c^	8.10 ± 0.18 ^a^	7.37 ± 0.47 ^b^	1.49 × 10^−5^
PE (16:0p/22:4)	PE	9.72 ± 0.08 ^a^	8.92 ± 0.06 ^b^	8.76 ± 0.04 ^c^	3.27 × 10^−7^
PE (18:1e/22:6)		7.51 ± 0.12 ^c^	8.35 ± 0.22 ^a^	8.09 ± 0.16 ^b^	9.23 × 10^−5^
PE (18:0/18:2)		8.80 ± 0.09 ^a^	7.78 ± 0.41 ^b^	7.05 ± 0.61 ^c^	0.0004913
PE (16:1/18:2)		6.88 ± 0.15 ^c^	7.81 ± 0.12 ^a^	7.40 ± 0.20 ^b^	2.12 × 10^−5^
PE (16:0p/18:3)		6.98 ± 0.36 ^b^	7.78 ± 0.32 ^a^	8.14 ± 0.17 ^a^	0.001239
PE (22:5/18:2)		7.09 ± 0.17 ^a^	6.50 ± 0.07 ^b^	5.95 ± 0.23 ^c^	0.0001772
PE (17:1/16:0)		5.56 ± 0.02 ^c^	6.76 ± 0.17 ^a^	6.14 ± 0.23 ^b^	2.15 × 10^−5^
PE (14:0/20:4)		5.69 ± 0.02 ^c^	6.50 ± 0.04 ^a^	6.17 ± 0.21 ^b^	5.62 × 10^−8^
PE (16:1/20:5)		5.36 ± 0.02 ^c^	6.50 ± 0.17 ^a^	6.02 ± 0.34 ^b^	2.79 × 10^−5^
PE (16:0/16:1)		5.16 ± 0.02 ^c^	6.40 ± 0.43 ^a^	5.88 ± 0.33 ^b^	0.0006704
PE (12:1e/20:4)		5.21 ± 0.02 ^c^	6.29 ± 0.26 ^a^	5.78 ± 0.32 ^b^	0.0002577
PG (18:1/14:0)	PG	5.21 ± 0.02 ^c^	5.91 ± 0.35 ^b^	6.69 ± 0.37 ^a^	0.00028
PS (18:2/21:0)	PS	8.37 ± 0.02 ^c^	9.23 ± 0.09 ^a^	8.88 ± 0.19 ^b^	4.36 × 10^−6^
PS (18:2/21:1)		7.56 ± 0.02 ^c^	9.09 ± 0.12 ^a^	8.20 ± 0.36 ^b^	1.30 × 10^−6^
PS (19:0/18:2)		7.25 ± 0.02 ^c^	8.57 ± 0.11 ^a^	7.87 ± 0.25 ^b^	1.32 × 10^−6^
PS (20:2/22:6)		6.11 ± 0.02 ^c^	6.97 ± 0.30 ^a^	6.64 ± 0.20 ^b^	0.0004723
PS (18:3/21:1)		5.40 ± 0.02 ^c^	7.65 ± 0.20 ^a^	6.56 ± 0.52 ^b^	2.46 × 10^−6^
PS (20:3/20:5)		4.99 ± 0.02 ^c^	7.04 ± 0.17 ^a^	6.23 ± 0.62 ^b^	1.72 × 10^−6^
SM (d14:0/18:1)	SM	7.20 ± 0.06 ^c^	8.52 ± 0.06 ^a^	8.30 ± 0.04 ^b^	5.25 × 10^−9^
SM (d20:0/24:5)		8.87 ± 0.06 ^a^	8.21 ± 0.05 ^b^	7.93 ± 0.07 ^c^	9.03 × 10^−8^
SM (d18:2/24:0)		6.75 ± 0.02 ^c^	8.17 ± 0.08 ^a^	7.51 ± 0.44 ^b^	2.72 × 10^−7^
SM (d18:1/20:3)		8.32 ± 0.07 ^a^	6.50 ± 0.14 ^c^	7.18 ± 0.31 ^b^	5.67 × 10^−7^
S1P (t17:0)	S1P	5.54 ± 0.05 ^c^	7.14 ± 0.68 ^a^	6.15 ± 0.43 ^b^	0.003374
TG (18:1/18:1/20:3)	TG	8.03 ± 0.32 ^b^	8.85 ± 0.05 ^a^	9.10 ± 0.10 ^a^	0.0006475
TG (16:0/10:0/14:0)		6.85 ± 0.29 ^c^	9.29 ± 0.25 ^a^	8.90 ± 0.16 ^b^	5.61 × 10^−6^
TG (16:0/10:0/18:2)		8.72 ± 0.44 ^a^	7.92 ± 0.28 ^b^	7.52 ± 0.33 ^b^	0.003706
TG (17:0/18:1/22:6)		7.09 ± 0.45 ^b^	8.35 ± 0.30 ^a^	8.68 ± 0.17 ^a^	0.0006787
TG (15:0/16:0/17:0)		7.46 ± 0.26 ^b^	8.29 ± 0.20 ^a^	8.03 ± 0.07 ^a^	0.003464
TG (10:0/18:1/18:3)		7.38 ± 0.37 ^b^	8.35 ± 0.21 ^a^	8.04 ± 0.24 ^a^	0.003261
TG (9:0/9:0/18:1)		7.30 ± 0.16 ^b^	8.01 ± 0.05 ^a^	8.13 ± 0.04 ^a^	4.77 × 10^−5^
TG (16:0/6:0/18:2)		7.07 ± 0.31 ^b^	8.23 ± 0.26 ^a^	7.92 ± 0.16 ^a^	0.001102
TG (10:0/10:0/18:1)		6.99 ± 0.49 ^c^	8.31 ± 0.25 ^a^	7.68 ± 0.24 ^b^	0.001897
TG (10:0/14:0/14:0)		6.44 ± 0.05 ^c^	8.49 ± 0.47 ^a^	7.14 ± 0.62 ^b^	0.0004872
TG (16:0e/18:0/22:5)		7.89 ± 0.24 ^a^	7.16 ± 0.56 ^b^	6.41 ± 0.52 ^c^	0.002177
TG (10:0/12:0/14:0)		6.04 ± 0.80 ^b^	7.88 ± 0.35 ^a^	7.20 ± 0.30 ^a^	0.003386
TG (18:3e/11:1/22:4)		5.77 ± 0.05 ^c^	7.11 ± 0.65 ^b^	7.93 ± 0.31 ^a^	4.88 × 10^−5^
TG (18:1/11:2/20:4)		5.87 ± 0.05 ^c^	7.56 ± 0.38 ^a^	6.82 ± 0.47 ^b^	0.0003313
TG (4:0/16:0/16:0)		5.93 ± 0.33 ^c^	7.27 ± 0.12 ^a^	6.60 ± 0.19 ^b^	0.0001132
TG (4:0/17:1/18:1)		5.47 ± 0.05 ^c^	7.51 ± 0.17 ^a^	6.11 ± 0.50 ^b^	1.95 × 10^−6^

Values with different superscript letters within the same row indicate significant differences between groups.

## Data Availability

The original contributions presented in this study are included in the article/supplementary material. Further inquiries can be directed to the corresponding author.

## Supplementary Materials

The following supporting information can be downloaded at: https://www.mdpi.com/article/10.3390/foods15162777/s1, Figure S1: Sample correlation analysis; Figure S2: Permutation test of PLS-DA.
