# Supplementary material for: Breed-Associated Differences in Longissimus Dorsi Muscle Lipid Composition in Saanen Dairy Goat, Shaanbei White Cashmere Goat, and Tan Sheep, with Implications for Flavour Formation
Source: Foods. 2026 Aug 7;15(16):2777. doi: 10.3390/foods15162777 (PMC13512775; doi:10.3390/foods15162777)

Figure S1. Sample correlation analysis.

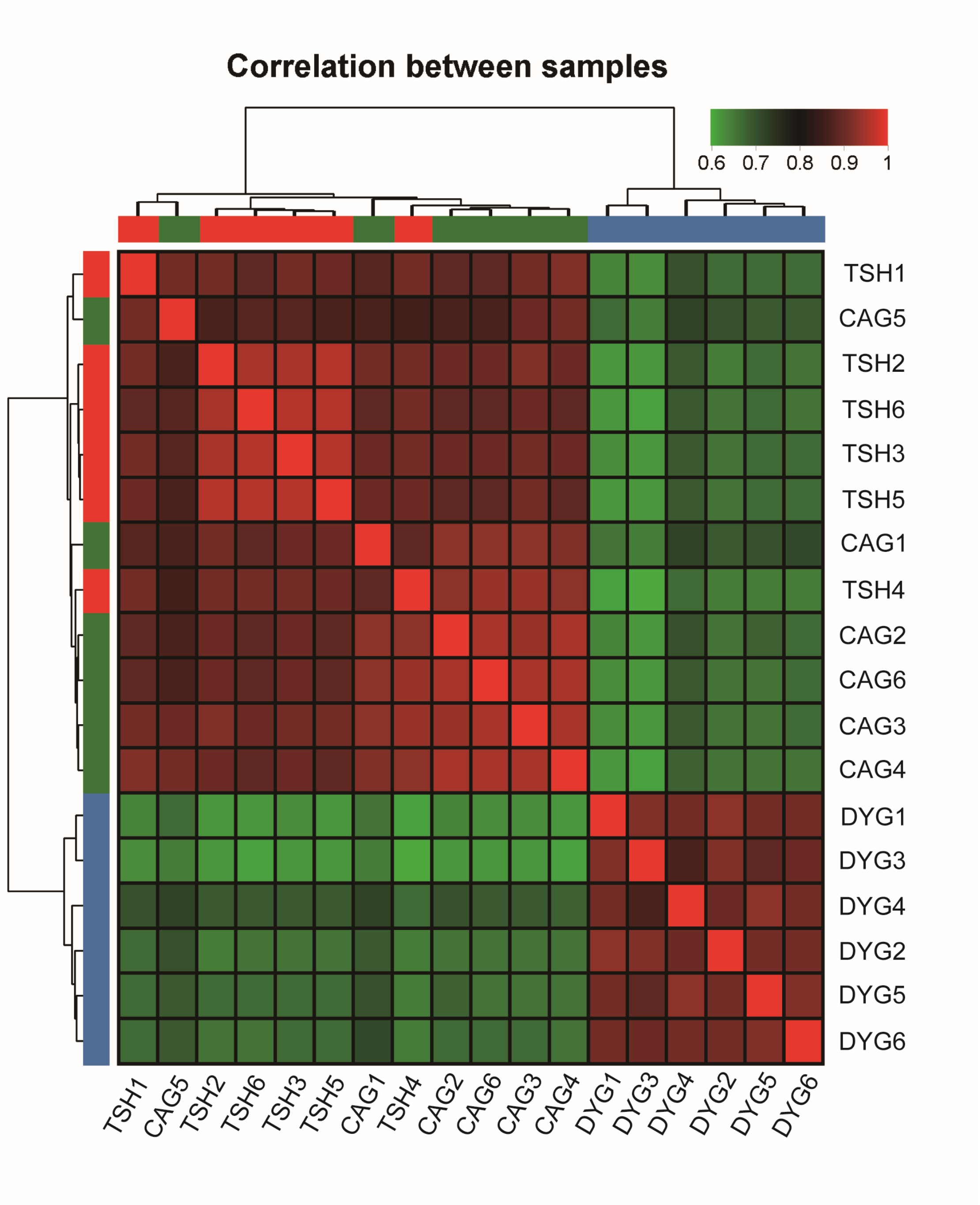

Figure S2. Permutation test of PLS-DA. (a) Permutation test in positive ion mode; (b) Permutation test in negative ion mode.

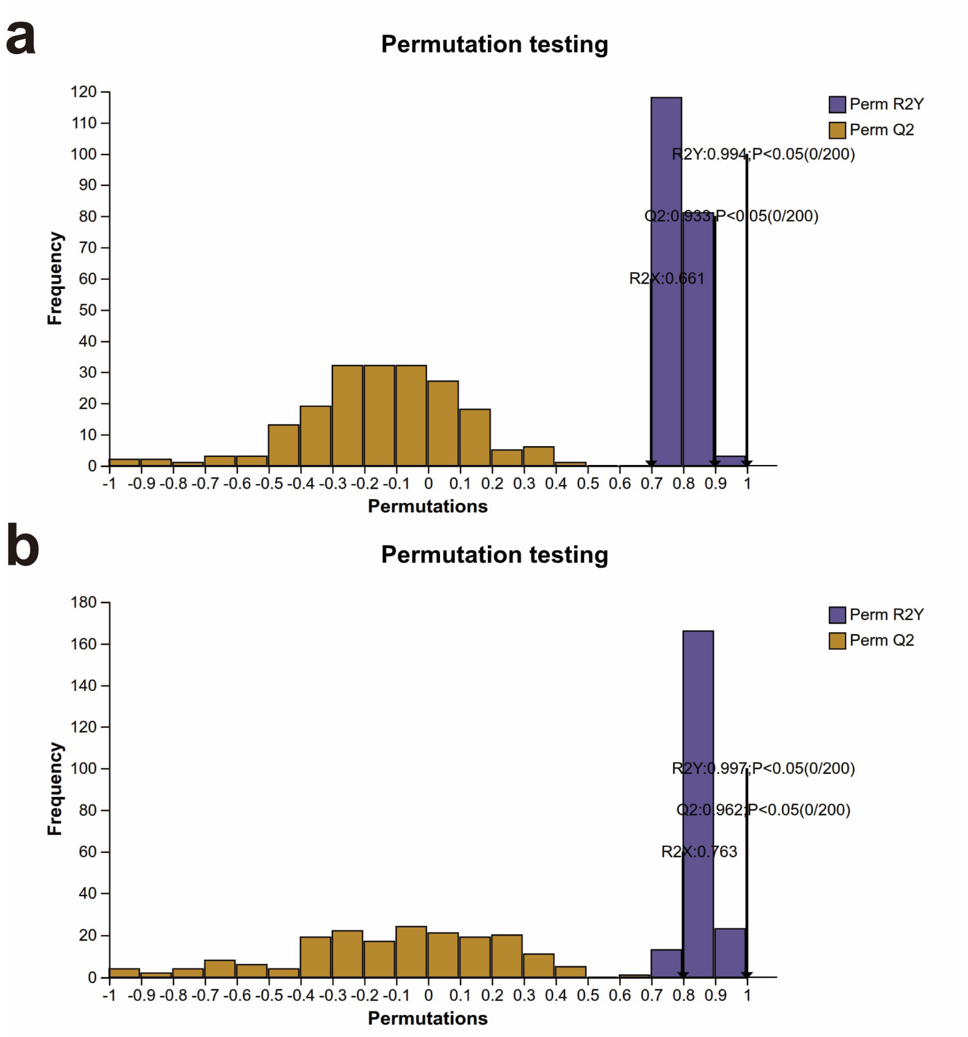

Supplement: Supplementary file 1 [file foods-15-02777-s001.zip › foods-4397274-supplementary.pdf]
